# Anodal tDCS and High-Frequency tRNS Targeting the Occipitotemporal Cortex Do Not Always Enhance Face Perception

**DOI:** 10.3389/fnins.2019.00078

**Published:** 2019-02-12

**Authors:** Megan L. Willis, Andrea I. Costantino, Michael. A. Nitsche, Romina Palermo, Davide Rivolta

**Affiliations:** ^1^School of Psychology, ARC Centre for Excellence in Cognition and Its Disorders, Australian Catholic University, Sydney, NSW, Australia; ^2^School of Psychology, University of East London, London, United Kingdom; ^3^Department of Psychology and Neuroscience, Leibniz Research Center for Working Environment and Human Factors, Dortmund, Germany; ^4^Department of Neurology, University Medical Hospital Bergmannsheil, Bochum, Germany; ^5^School of Psychological Science, ARC Centre for Excellence in Cognition and Its Disorders, University of Western Australia, Perth, WA, Australia; ^6^Department of Education, Psychology and Communication, University of Bari Aldo Moro, Bari, Italy

**Keywords:** tDCS, tRNS, transcranial electrical stimulation, mild brain stimulation, face perception, facial expression perception, object perception

## Abstract

There has been increasing interest in the utility of transcranial electrical stimulation as a tool to enhance cognitive abilities. In the domain of face perception, enhancements have been reported for both transcranial direct current stimulation (tDCS) and high-frequency transcranial random noise stimulation (tRNS) targeting the occipitotemporal cortex. In a series of two experiments, we attempted to replicate these findings for face identity perception, and extend on previous studies, to determine if similar enhancements are also observed for object and facial expression perception. In Experiment 1, using a single blind, between-subjects design in healthy volunteers (*N* = 53), we examined whether anodal tDCS over the occipitotemporal cortex enhanced performance on tasks involving perception of face identity, facial expression, and object stimuli, when compared to sham stimulation. We failed to replicate previous findings of enhanced performance on face and object perception, nor extend findings to facial expression perception. In Experiment 2, using a single blind, between-subjects design (*N* = 39), we examined the effect of high-frequency tRNS over the occipitotemporal cortex using the same three tasks employed in Experiment 1. We failed to replicate previous findings of enhanced face perception following high-frequency tRNS over the occipitotemporal cortex, relative to sham stimulation (although we used different stimulation parameters to that employed in a previous study). We also found no evidence of enhanced facial expression and object perception following high-frequency tRNS. The findings align with a growing body of studies that have failed to replicate previously reported enhancements following administration of tDCS and hint for different efficacy of, on first sight, related stimulation protocols. Future studies should explore the foundation of these differential effects in greater detail.

## Introduction

There has been a surge of research interest concerning individuals who experience difficulties in face recognition (for reviews, see Rivolta et al., 2013; Corrow et al., 2016). Face recognition impairments have been well documented across a range of populations, including individuals with congenital prosopagnosia (Behrmann and Avidan, 2005; Rivolta et al., 2012) and acquired prosopagnosia (Della Sala and Young, 2003; Barton, 2008), for whom face recognition difficulties often exist in the context of generally preserved object and facial expression recognition skills (Palermo et al., 2011). Face recognition difficulties have also been observed in clinical populations who experience a range of social cognitive difficulties, including individuals with conditions such as autism spectrum disorder (e.g., Tang et al., 2015), and schizophrenia (e.g., Green et al., 2015). Given that the capacity to accurately recognize the faces of other individuals facilitates effective social communication (Yardley et al., 2008), difficulties in face recognition present a considerable challenge for effective social functioning that has far reaching consequences on an individual’s interpersonal, educational, and occupational functioning. Developing effective treatments that can remediate face recognition deficits is therefore an important research topic (Barbieri et al., 2016).

In recent years, considerable interest has been emerging surrounding the potential utility of non-invasive transcranial electrical stimulation techniques to enhance cognitive and perceptual skills in humans (Kuo and Nitsche, 2012). One transcranial electrical stimulation technique, which has received the most research interest to date, is *transcranial direct current stimulation* (tDCS). The traditional tDCS setup involves the application of a weak electrical current (0.5–2 mA) to the scalp via two electrodes (i.e., the anode and cathode). The widespread assumption is that tDCS exerts its effects by modulating cortical excitability via subthreshold depolarization or hyperpolarization of resting membrane potentials, with anodal stimulation inducing excitatory effects via depolarization, and cathodal stimulation exerting inhibitory effects via hyperpolarization (Nitsche et al., 2008). The extent to which these changes in cortical excitability transfer to enhanced cognitive performance (following anodal stimulation) and diminished cognitive performance (following cathodal stimulation) is far from conclusive, with a notable lack of evidence to support the assertion that cathodal stimulation diminishes cognitive performance (e.g., Jacobson et al., 2012; Costantino et al., 2017).

To date, anodal tDCS has been found to lead to enhancements across different cognitive and perceptual domains, including visual perception, working memory, long term memory, attention, and executive functioning (for reviews, see Kuo and Nitsche, 2012; Dedoncker et al., 2016). Studies have also emerged providing evidence for enhancements in face recognition. Specifically, anodal tDCS targeting the right occipitotemporal cortex has been found to result in superior accuracy in face perception and memory for faces (Barbieri et al., 2016), and superior working memory for faces (Brunye et al., 2017).

Findings of enhanced face processing following anodal tDCS present an exciting advance in the field and hold promise for the utility of tDCS as a potential intervention for individuals with face processing difficulties. However, to date, these initial findings are yet to be replicated. The importance of replication in the field has been illustrated by emerging concerns regarding the replicability of tDCS effects, as indicated by a growing number of failures to replicate positive effects of tDCS (e.g., Koenigs et al., 2009; Vannorsdall et al., 2016), null findings (e.g., de Hollander et al., 2016; Nilsson et al., 2017), and meta-analyses providing heterogeneous evidence of tDCS efficacy in cognitive domains (e.g., Hill et al., 2016; Medina and Cason, 2017; Simonsmeier et al., 2018).

Thus, in the current study we conducted two experiments where we sought to replicate recent findings (Romanska et al., 2015; Barbieri et al., 2016) reporting enhanced face perception following transcranial electrical stimulation techniques. The primary aim of Experiment 1 was to replicate enhancements in face perception following anodal tDCS compared to sham stimulation, which were observed with a medium effect size and group sample sizes of 16 (Barbieri et al., 2016). Here, consistent with Barbieri et al. (2016) we employed a between-subjects design with a larger group sample size (see details below in section Materials and Methods) than that used in Barbieri et al. (2016) to investigate whether anodal tDCS targeting the right occipitotemporal cortex results in enhancements in face perception, compared to sham stimulation. We also employed the same face perception task as used in Barbieri et al. (2016). The task involves presentation of three unfamiliar faces, comprising two faces of the same identity and a third, different identity. The participant’s task is to identify which face is the odd-one-out (i.e., not the same identity).

In addition to investigating the effect of anodal tDCS on face perception, we also sought to investigate whether the effects of anodal tDCS extended to object perception and facial expression perception. There is evidence that anodal tDCS over the occipitotemporal cortex also leads to enhancements in object perception (Barbieri et al., 2016) when assessed using an odd-one-out task, equivalent in structure to the face perception task also employed in Barbieri et al. (2016). While studies have reported enhancements in facial expression recognition following anodal tDCS targeting the prefrontal cortex (Willis et al., 2015), as yet, no studies to our knowledge have investigated the effect of anodal tDCS targeting the occipitotemporal cortex on facial expression perception. There is emerging evidence that occipitotemporal brain regions are involved in a common stage of perceptual processing of both face identity and facial expression (Calder and Young, 2005; Palermo et al., 2013; Rhodes et al., 2015) thus leading to the prediction that enhancements following anodal tDCS over the occipitotemporal cortex will also be seen for facial expression perception. A comparison of the effects of tDCS across face identity, facial expression, and object perception tasks also has the potential to provide insight into the specificity of any observed tDCS effects that might be of conceptual importance for prominent models of face and facial expression recognition. That is, are the effects of tDCS specific to face identity or the face more broadly (i.e., identity and expression)?

Finally, given emerging recognition of the importance of inter-individual variability on the effects of transcranial electrical stimulation (for reviews, see Li et al., 2015; Fertonani and Miniussi, 2017), we also collected information on demographic variables (i.e., age, sex, race) and obtained measurements of IQ, and baseline face identity and facial expression recognition performance in order to confirm the absence of group differences in these variables, and control for relevant variables in the event of group differences.

In Experiment 2, we then sought to reproduce recent findings of enhanced face perception (Romanska et al., 2015), using another transcranial electrical stimulation technique, *high-frequency transcranial random noise stimulation* (tRNS). Consistent with findings of enhanced face perception by Barbieri et al. (2016), enhanced face perception has also been observed using high-frequency tRNS (Romanska et al., 2015). Compared to tDCS, high-frequency tRNS involves applying an alternating current over the cortex at random frequencies within a broad spectrum (e.g., 100–640 Hz; Terney et al., 2008). High-frequency tRNS applied to the occipitotemporal cortex bilaterally, has been found to lead to more accurate performance in face perception as assessed on the Cambridge Face Perception task, relative to sham stimulation (Romanska et al., 2015). High-frequency tRNS is thought to lead to enhanced excitability at both electrode sites (Pirulli et al., 2016), this is in contrast to tDCS, where the anode induces excitatory effects and the cathode induces inhibitory effects (at least as investigated on the motor cortex) (Nitsche and Paulus, 2000). The transfer of tDCS induced excitatory/inhibitory effects to cognitive performance is not consistently observed, which has been postulated to reflect the widespread brain networks that underpin cognitive tasks (Jacobson et al., 2012).

Since cognitive and perceptual tasks rely on a widespread network of neural regions, there is the potential for the cathode to induce inhibitory effects on cognitive performance following tDCS. As high-frequency tRNS is thought to exert excitatory effects under both electrode sites (Pirulli et al., 2016), it may be more effective at enhancing face perception than anodal tDCS. As such, we were interested in determining if we could replicate the previously reported effect of high-frequency tRNS enhancing face perception (Romanska et al., 2015). Here, this was examined using the same face perception task employed in Experiment 1 (and in Barbieri et al., 2016), in order to enable comparison across Experiment 1 (anodal tDCS) and Experiment 2 (high-frequency tRNS). We used different stimulation parameters (i.e., current intensity and frequency range) to that utilized by Romanska et al. (2015), in order to facilitate comparison across Experiment 1 and 2, and as a result of technical limitations of the stimulation device in the current study. In line with the rationale outlined for Experiment 1, we also sought to extend the current experiment to also investigate the effect of high-frequency tRNS on facial expression and object perception.

## Experiment 1

The purpose of Experiment 1 was to compare the effect of anodal tDCS targeting the right occipitotemporal cortex, to sham stimulation, on comparable tasks assessing face identity, facial expression, and object perception. Based on previous findings of enhanced face and object perception following anodal tDCS over the occipitotemporal cortex (Barbieri et al., 2016), we expected to replicate these effects, with a medium effect size (η*_p_*^2^ = 0.20, Barbieri et al., 2016), and extend them to facial expression perception, given growing evidence for a common stage of processing in the perception of face identity and facial expression (Calder and Young, 2005; Palermo et al., 2013; Rhodes et al., 2015).

### Materials and Methods

#### Participants

The final sample comprised 53 (43 female) participants whose ages ranged from 18 to 49 (*M* = 23.09, *SD* = 6.57). Participants were undergraduate students and members of the wider community. All were right-handed, fluent in English, and had normal or corrected-to-normal visual acuity. Two participants were excluded as a computer malfunction resulted in incomplete data. A further three participants were excluded due to suspected congenital prosopagnosia, as assessed by the Cambridge Face Memory Task (see description in Baseline Measures section below; i.e., score < 38, see norms in Bowles et al., 2009). Participants attended the lab for one session and were randomly assigned to either active tDCS (*n* = 29) or sham tDCS (*n* = 24).

#### Baseline Measures

Prior to stimulation, participants completed several tasks to determine if there were any baseline group differences in face identity recognition, facial expression recognition ability and intelligence that may account for performance on the measures of interest.

#### Test of Premorbid Function (TOPF)

The TOPF provides an estimate of intellectual functioning (Wechsler, 2009). In this task, participants are asked to read a list of up to 70 atypical grapheme words and are required to pronounce each word as accurately as possible. Participant’s total raw score (correct number of pronunciations) was converted into a standard score with reference to age-norms.

#### Cambridge Face Memory Task (CFMT; Duchaine and Nakayama, 2006)

The CFMT was used to assess face identity recognition for two purposes. First, to screen for participants with suspected congenital prosopagnosia, exclusions are noted above in the Participants section. Second, to provide a baseline measure of face identity recognition ability. The CFMT assesses memory for unfamiliar faces. Participants are shown six unfamiliar faces, which they are required to learn for recognition across three different viewing conditions: (1) the same face images; (2) the same faces in different images (i.e., different viewpoint and/or lighting); (3) the same faces in different images covered with visual noise. The task comprises 72 trials in total. Accuracy rates were recorded. For a more detailed description of the methodology, see Duchaine and Nakayama (2006).

#### Emotion Hexagon (Young et al., 2002)

The Emotion Hexagon task was used to provide a baseline measure of facial expression recognition ability. The task involves presentation of a series of grayscale images of an individual displaying six basic emotions that are blended together based on a confusion matrix (angry – disgusted; disgusted – sad; sad – fearful; fearful – surprised; surprised – happy; happy – angry) in five stages (90/10; 70/30; 50/50; 30/70; 10/90). Participants viewed each photograph for 5 s, and were given an unlimited time to select by mouse click the label (angry, disgusted, happy, fearful, sad, surprised), which best depicted the emotion displayed in the image. Participants completed one practice block, followed by five experimental blocks, each comprising 30 trials. The data for trials containing equal (i.e., 50/50) blends were excluded from analyses, thus resulting in a maximum accuracy score of 120. The task was administered using SuperLab 5 (Cedrus Corp.) experimental software.

### Experimental Tasks

#### Face Identity

The face identity task described in Barbieri et al. (2016) was used to measure face identity perception. This task is adapted from a task originally developed by Barense et al. (2011). Three grayscale images of Caucasian unfamiliar faces were presented simultaneously on each trial. Two images were of the same face identity presented in different angles, while the third image was of a different face identity. Participants were asked to specify which face identity was different from the other two, by pressing the equivalent number on the keyboard (i.e., 1, 2, or 3) as fast and accurately as possible. The images were presented for 4 s. If the participant did not respond during the presentation window, the response was coded as incorrect. Each trial was followed by an inter-trial interval of 2 s. Participants completed four practice trials (with stimuli different from those used in the experimental trials). A total of 70 trials were presented in the task (this was a smaller subset of trials compared to 81 used in Barbieri et al., which were selected in order to have an equivalent number of trials across the three tasks that were equated on average difficulty based on pilot data), with participants given the opportunity to take a short break after 35 trials. The task was administered using SuperLab 5 (Cedrus Corp.) experimental software. Percentage accuracy and reaction times (RT) were recorded.

#### Facial Expression

The facial expression perception task was adapted from the emotion-matching task described in Palermo et al. (2013). The task was created to have the same structure as the face identity task. Three grayscale images of different Caucasian faces were presented on each trial. Two faces depicted the same facial expression, while the third image was displaying a different facial expression. Participants were asked to indicate which face was portraying a different expression from the other two. The procedure was otherwise identical to the face identity perception task.

#### Object

The object perception task followed the same structure as the face identity and facial expression tasks. In this task, objects, such as fruit and furniture, were used as stimuli. Stimuli were the same as those used in Barbieri et al. (2016), originally sourced from Barense et al. (2011). In this task, participants were required to identify which object was different from the other two. The procedure was otherwise identical to the face identity and facial expression perception tasks.

#### Stimulation

The protocol for tDCS was similar to that employed by Barbieri et al. (2016). The anode was positioned over the right occipitotemporal cortex, equivalent to location PO8 (positioned according to the international 10–20 EEG system), with the cathode positioned over the left prefrontal cortex (equivalent to location FP1). In the active tDCS condition, a constant current flow of 1.5 mA was applied through a pair of saline soaked sponge electrodes (25 cm^2^; current density 0.080 mA/cm^2^) for 20 min via a battery-driven constant current stimulator (Neuroelectrics^©^, Barcelona, Spain). The stimulation commenced after 10 s ramp-up time. In the sham tDCS condition, stimulation was administered for the first and last 10 s of the 20 min session. Participants were blind to condition (i.e., active or sham), but the administrator was not.

#### Procedure

This study was designed and conducted in accordance with the Declaration of Helsinki and approved by the Human Research Ethics Committees of the Australian Catholic University and University of East London. All participants provided written informed consent. Participants attended the lab for one session, lasting approximately 90 min. Prior to participation, all participants were screened to ensure there were no contraindications (i.e., metal in the scalp, history of migraines etc.) for tDCS. All participants provided written informed consent at the beginning of the session. The baseline tasks were completed in a fixed order: (1) TOPF; (2) Emotion Hexagon; (3) CFMT. Stimulation (active or sham) was then administered for 20 min. During this period, the participant was seated comfortably on the chair. The experimental tasks were completed immediately following stimulation (i.e., offline, as per Barbieri et al., 2016). To control for order effects, the three experimental tasks (i.e., face identity, facial expression, and object perception) were completed in a counterbalanced order across participants. All tasks were administered on a 13-inch MacBook Pro laptop.

### Results

#### Participant Characteristics

Participant characteristics and baseline performance are reported in Table 1. There was no evidence to suggest that the two groups differed with respect to demographic variables, IQ, or baseline face identity and facial expression recognition performance. A series of independent samples *t*-tests comparing the two groups (i.e., active tDCS, sham tDCS) confirmed that there was no significant difference with respect to age, *t*(51) = 0.01, *p* = 0.991, or IQ, *t*(51) = 0.10, *p* = 0.920. Furthermore, no significant difference emerged between the two groups in baseline face identity performance as measured by the CFMT, *t*(51) = 0.27, *p* = 0.787, and baseline facial expression recognition performance as measured with the Emotion Hexagon task, *t*(51) = 0.23, *p* = 0.817. Fisher’s exact test confirmed there was no significant difference in the distribution of males and females, or Caucasians and non-Caucasians, across the two groups, *p*s ≥ 0.482.

**Table 1:** Demographics and baseline task performance for groups in experiment 1, including demographic data from Barbieri et al. (2016) for comparison.

	Experiment 1		Barbieri et al. (2016)	
	Active tDCS	Sham tDCS	Active tDCS	Sham tDCS
*n*	29	24	16	16
Sex	25F:4M	18F:6M	11F:5M	10F:6M
Caucasian (*n*)	14	13	–	–
Age (years)	23.10 (6.52)	23.08 (6.77)	28.88 (6.05)	25.13 (6.74)
IQ	98.07 (11.21)	98.42 (13.77)	–	–
CFMT^a^	56.48 (8.33)	55.83 (9.05)	–	–
EH^b^	100.69 (10.75)	100.04 (9.24)	–	–

*Note. Standard deviation is presented in brackets; F, female; M, male; EH, Emotion Hexagon; CFMT, Cambridge Face Memory Task.^*a*^ Maximum score is 72.^*b*^ Maximum score is 120*.

#### Statistical Analyses

The primary analysis was a two-way mixed model analysis of variance (ANOVA) with the between-subjects factor of *tDCS condition* (active, sham) and the within-subjects factor of *task* (face identity, facial expression, object), which was performed on the dependent measures of percentage accuracy and mean reaction time for correct trials. The Greenhouse-Geisser epsilon adjusted value is reported where the sphericity assumption was violated. Significant main effects and interactions were investigated by performing Bonferroni corrected follow-up comparisons. In order to provide more informative statements regarding evidence in support of our hypotheses, relative to the null hypotheses, we also performed the equivalent analyses using Bayesian hypothesis testing in JASP (JASP Team, 2018). Thus, in addition to conventional ANOVA statistics, we also report the Bayes factors (BF_10_) accompanied by an estimate of error, expressed as a percentage (see Wagenmakers et al., 2018a,b, for information on the advantages of Bayesian hypothesis testing and performing and interpreting Bayesian hypothesis testing using JASP software). Please refer to Supplementary Material for the analyzed data set.

### Primary Analysis

#### Accuracy

**FIGURE 1:**
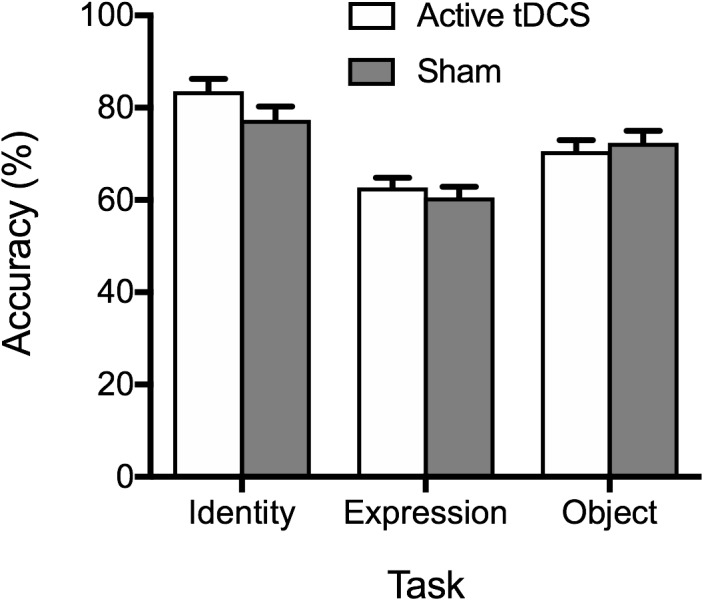
Mean percentage accuracy on the identity, expression, and object tasks for the active tDCS and sham groups. Standard error bars are displayed in this and all subsequent figures.

Mean percentage accuracy is displayed in Figure 1. Analysis of accuracy data provided no evidence of superior accuracy following active tDCS (compared to sham) as indicated by a non-significant main effect of tDCS condition, *F*(1, 51) = 0.68, *p* = 0.414, η_p_^2^ = 0.01, BF_10_ = 0.28 ± 0.71%. The tDCS Condition × Task interaction was also non-significant, *F*(2, 102) = 1.97, *p* = 0.145, η_p_^2^ = 0.04, BF_10_ = 0.52 ± 2.72%. A significant main effect of task emerged, *F*(2, 102) = 43.56, *p* < 0.001, η_p_^2^ = 0.46, BF_10_ > 100 ± 0.84%, reflecting superior performance on the face identity task, compared to the facial expression and object tasks (*p*s < 0.001). Performance was also more accurate on the object task, compared to the facial expression task (*p* < 0.001).

#### RT

**FIGURE 2:**
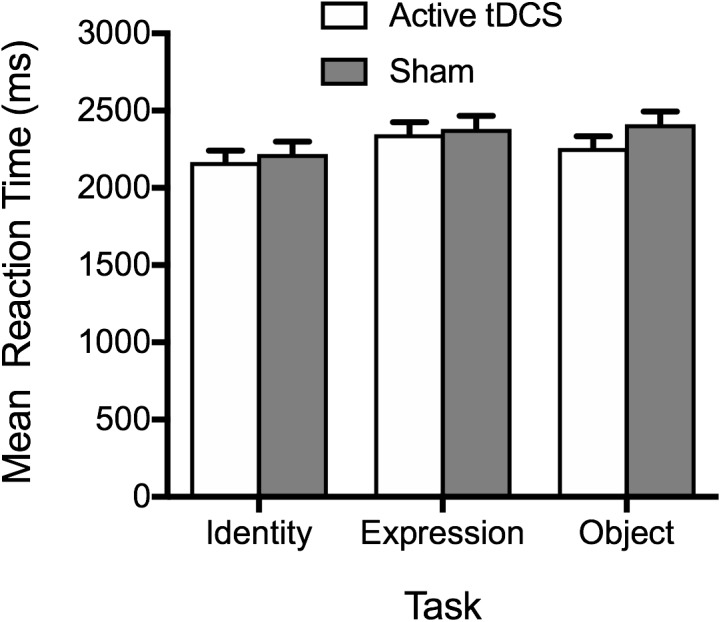
Mean correct reaction times on the identity, expression, and object tasks for the active tDCS and sham groups.

In line with accuracy data, analysis of RT data provided no evidence of active tDCS stimulation leading to faster RTs, relative to sham stimulation (see Figure 2). The main effect of tDCS condition was non-significant, *F*(1, 51) = 0.71, *p* = 0.404, η_p_^2^ = 0.01, BF_10_ = 0.42 ± 0.62%, as was the tDCS Condition × Task interaction, *F*(2, 102) = 0.73, *p* = 0.486, η_p_^2^ = 0.01, BF_10_ = 0.19 ± 2.43%. As with accuracy, a significant main effect of task emerged, *F*(2, 102) = 5.88, *p* = 0.004, η_p_^2^ = 0.10, BF_10_ = 8.27 ± 1.04%, reflecting faster performance on the face identity task, compared to the facial expression and object tasks (*p*s ≤ 0.049). There was no significant difference between RTs on the facial expression and object tasks (*p* = 1.00).

### Summary of Findings

In summary, we failed to replicate the findings of Barbieri et al. (2016), which demonstrated superior face and object perception following anodal tDCS targeting the right occipitotemporal cortex. While the pattern of results was in the predicted direction (i.e., faster RT and superior performance following anodal tDCS compared to sham), the observed effect size in the current study was small (contrasted with a medium effect size observed in Barbieri and colleagues’ study) and performance was not significantly enhanced following anodal tDCS, compared to sham stimulation, in analyses of performance accuracy or reaction time. This was despite our group sample sizes (active tDCS = 29; sham = 24) being larger than the group sample sizes of 16 in Barbieri et al. (2016). Bayesian hypothesis testing aligned with the ANOVA findings, providing no evidence in support of our prediction that anodal tDCS would lead to enhancements in accuracy and RT, relative to sham stimulation. Specifically, interpretation of the Bayes Factors indicated that the results provide anecdotal to moderate evidence (across analyses of accuracy and RT data) for the null hypothesis.

## Experiment 2

The aim of Experiment 2 was to investigate whether, compared to sham stimulation, high-frequency tRNS over the occipitotemporal cortex enhanced performance on face identity, facial expression, and object perception using the same tasks employed in Experiment 1. Based on previous findings of enhanced face perception following high-frequency tRNS targeting the occipitotemporal cortex (Romanska et al., 2015), we expected to replicate these effects, and extend them to facial expression and object perception.

### Materials and Methods

#### Participants

The final sample comprised 39 (26 female) participants whose ages ranged from 18 to 54 (*M* = 25.36, *SD* = 8.05) who were undergraduate students and members of the wider community. As in Experiment 1, participants were right-handed, fluent in English, had normal or corrected-to-normal visual acuity. The data of two participants was excluded due to suspected congenital prosopagnosia (as per criteria defined in Experiment 1). Participants attended the lab for one session and were randomly assigned to either active tRNS (*n* = 19) or sham tRNS (*n* = 20).

#### Baseline Measures

As in Experiment 1, prior to stimulation, participants completed the TOPF, CFMT, and Emotion Hexagon to determine if there were any baseline group differences in intelligence, face identity recognition, and facial expression recognition ability that may account for performance on the measures of interest.

#### Experimental Tasks

The tasks employed in the current experiment were identical to that employed in Experiment 1.

#### Stimulation

Administration of high-frequency tRNS and sham tRNS was largely as described above in Experiment 1 for tDCS, with the exception that the electrodes were positioned bilaterally to target the left and right occipitotemporal cortex at PO7 and PO8, respectively, with an alternating (100–500 Hz) 1.5 mA peak-to-peak current applied without a DC offset.

#### Procedure

The procedure followed that described in Experiment 1, except where otherwise noted above.

### Results

#### Participant Characteristics

Participant characteristics and baseline performance are reported in Table 2. As in Experiment 1, there were no group differences with respect to demographic variables, IQ, or baseline face identity and facial expression recognition performance. This was confirmed by a series of independent samples t-tests comparing the two tRNS groups (i.e., active tRNS, sham tRNS) for age, *t*(37) = 0.68, *p* = 0.502, IQ, *t*(37) = 0.05, *p* = 0.960, baseline face identity performance as measured by the CFMT, *t*(37) = 0.34, *p* = 0.736, and baseline facial expression recognition performance measured by the Emotion Hexagon, *t*(37) = 0.10, *p* = 0.923. Fisher’s exact test also confirmed there was no significant difference in the distribution of males and females, or Caucasians and non-Caucasians, across the two groups, *p*s ≥ 0.741.

**Table 2:** Demographics and baseline task performance for groups in Experiment 2, including demographic data from Romanska et al. (2015) for comparison.

	Experiment 2		Romanska et al. (2015)	
	Active tRNS	Sham tRNS	Active tRNS	Sham tRNS
*n*	19	20	18	18
Sex	12F:7M	14F:6M	11F:7M	12F:6M
Caucasian (*n*)	10	11	–	–
Age (years)	26.26 (7.67)	24.50 (8.51)	26.80 (3.50)	27.20 (5.10)
IQ	100.53 (13.87)	100.30 (13.84)	–	–
CFMT^a^	54.05 (7.66)	53.10 (9.69)	–	–
EH^b^	99.74 (14.32)	100.15 (12.00)	–	–

*Standard deviation is presented in brackets; F, female; M, male; EH, Emotion Hexagon; CFMT, Cambridge Face Memory Task.^*a*^ Maximum score is 72.^*b*^ Maximum score is 120*.

#### Statistical Analyses

The statistical analyses employed here were consistent with that described in Experiment 1. That is, for the dependent measures of percentage accuracy and mean reaction time for correct trials, we performed two-way mixed model ANOVAs with the between-subjects factor of *tRNS condition* (active, sham) and the within-subjects factor of *task*. We again conducted Bayesian hypothesis testing in order to provide more informative statements regarding evidence in support of our hypotheses. Please refer to Supplementary Materials for the analyzed data set.

### Primary Analysis

#### Accuracy

**FIGURE 3:**
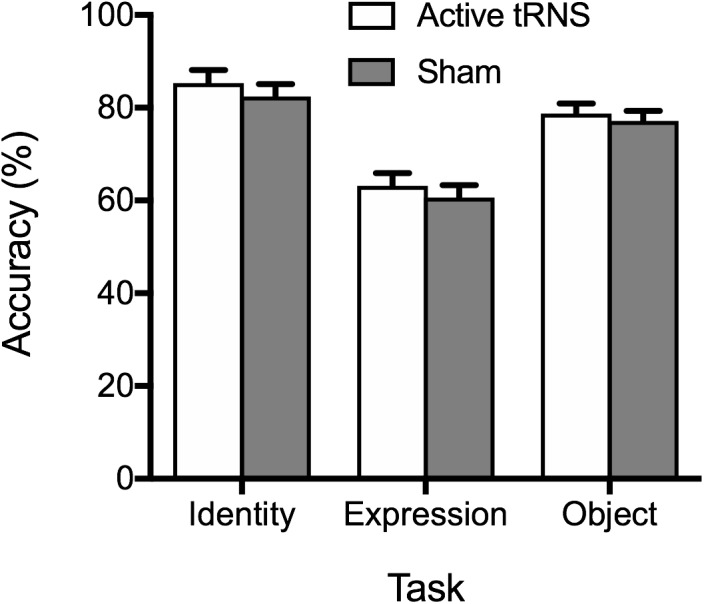
Mean percentage accuracy on the identity, expression, and object tasks for the active tRNS and sham groups.

Consistent with Experiment 1, analysis of accuracy data failed to provide evidence of superior accuracy following stimulation (see Figure 3), as indicated by a non-significant main effect of tRNS condition, *F*(1, 37) = 0.64, *p* = 0.428, η_p_^2^ = 0.02, BF_10_ = 0.30 ± 0.75%. The tRNS Condition × Task interaction was also non-significant, *F*(2, 74) = 0.07, *p* = 0.936, η_p_^2^ = 0.00, BF_10_ = 0.14 ± 1.75%. As with Experiment 1, a significant main effect of task emerged, *F*(2, 74) = 72.81, *p* < 0.001, η_p_^2^ = 0.66, BF_10_ > 100 ± 1.38%, reflecting superior performance on the face identity task, compared to the facial expression and object tasks (*p*s ≤ 0.018). Performance was also more accurate on the object task compared to the facial expression task (*p* < 0.001).

#### RT

**FIGURE 4:**
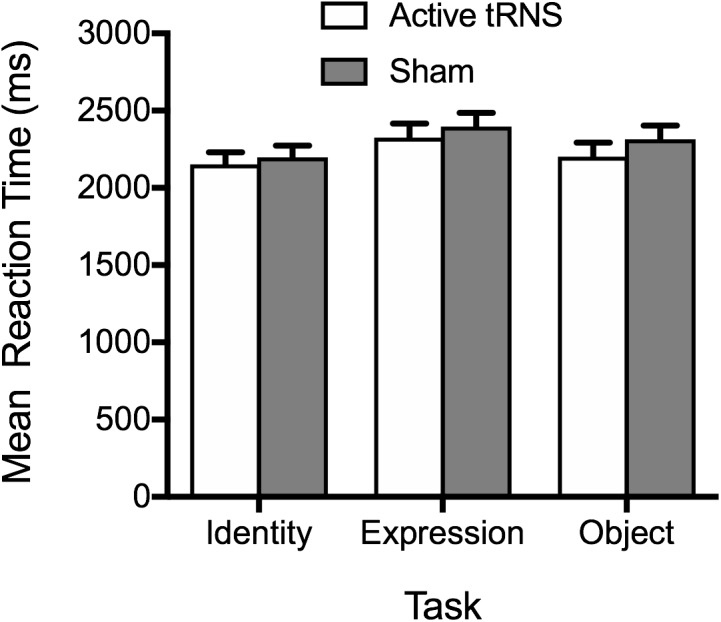
Mean correct reaction times on the identity, expression, and object tasks for the active tRNS and sham groups.

The pattern of results aligned with accuracy data (see Figure 4), providing no evidence for stimulation enhancing performance. The main effect of tRNS condition was non-significant, *F*(1, 37) = 0.48, *p* = 0.492, η_p_^2^ = 0.01, BF_10_ = 0.50 ± 0.86%, along with the tRNS Condition × Task interaction, *F*(2, 74) = 0.26, *p* = 0.768, η_p_^2^ = 0.01, BF_10_ = 0.16 ± 4.66%. A significant main effect of task again emerged, *F*(2, 74) = 8.47, *p* < 0.001, η_p_^2^ = 0.19, BF_10_ = 64.63 ± 0.64%, reflecting faster performance on the face identity task, compared to the facial expression tasks, *p* = 0.001. There was no significant difference between RTs on the object task compared to face identity and facial expression task, after Bonferroni correction, *ps* ≥ 0.104.

### Summary of Findings

In summary, as in Experiment 1, the pattern of results was in the predicted direction (i.e., faster RT and superior performance following high-frequency tRNS compared to sham), however, the effect size was small, and performance was not significantly enhanced following high-frequency tRNS, compared to sham stimulation, in analyses of performance accuracy and reaction time for any task. The results of Bayesian hypothesis testing further aligned with the results providing no evidence for an effect of the tRNS condition, or interaction with task. Indeed, interpretation of Bayes Factors provided anecdotal to moderate evidence for the null model. There were no group differences on any demographic variables, IQ, or baseline performance, suggesting that these variables were unlikely to account for a failure to find enhanced face perception following high-frequency tRNS over the occipitotemporal cortex. Thus, despite group sample sizes being of equivalent size to Romanska et al. (2015), we failed to replicate their finding of enhanced face perception following high-frequency tRNS targeting the occipitotemporal cortex in the current study. In the General Discussion, we discuss in detail important differences in the stimulus parameters used in the current study, to those employed in Romanska et al. (2015), that may account for the failure to replicate their findings.

## General Discussion

The primary aim of the current study was to replicate previous research findings of enhanced face perception using two transcranial electrical stimulation techniques, anodal tDCS and high-frequency tRNS, both targeting the occipitotemporal cortex. As a secondary aim, we sought to determine if such enhancement also extended to object and facial expression perception.

In Experiment 1, we found no evidence of enhanced face perception following anodal tDCS targeting the right occipitotemporal cortex. Moreover, there was no evidence of enhanced object or facial expression perception following anodal tDCS. There was no evidence of group differences in demographic variables and baseline performance. In Experiment 2, we also failed to observe the predicted effect of enhanced face perception following high-frequency tRNS targeting the occipitotemporal cortex, with no evidence of any enhancement also observed for the facial expression and object perception tasks.

The findings of Experiment 1 failed to replicate those reported by Barbieri et al. (2016) of enhanced perception following anodal tDCS over the right occipitotemporal cortex. Of importance, this was despite utilizing the same face perception task that Barbieri and colleagues employed, as well as recruiting a larger sample size in the current study. While the same face perception task used by Barbieri and colleagues was utilized in the current study, one minor methodological change was incorporated into the current study that may be important. Specifically, the current study comprised a smaller number of trials of 70, compared to 81 trials in Barbieri et al. The number of trials was reduced in an attempt to create three tasks of equivalent length and difficulty to assess face identity, facial expression and object perception. Despite a pilot study guiding selection of items to equate difficulty of the tasks, it was evident that in the current cohort of participants there were marked differences in the difficulty of the three tasks, with the face perception task associated with superior performance, compared to the object and facial expression tasks. A comparison of performance accuracy on the face perception task in the current study, relative to Barbieri et al. (2016) suggests largely comparable performance across the two sham groups (*M* = 77 current study, *M* ≈ 75 in Barbieri), with a slightly higher mean in the current study. It is, however, possible that reducing the number of trials in the object and face perception tasks in the current study may have reduced the tasks’ sensitivity, when compared to Barbieri et al., which may in turn have contributed to our failed attempt to replicate their findings.

The results of Experiment 2 do not align with findings of Romanska et al. (2015), who observed enhanced face perception following high-frequency tRNS over the occipitotemporal cortex. Experiment 2 was not a direct replication Romanska et al. (2015). As such there are several noteworthy differences in methodology that could account for the divergent findings. First, the face perception tasks employed across the two studies were different. While Romanska and colleagues employed the Cambridge Face Perception Test (CFPT), we employed the same odd-one-out face perception task as used in Experiment 1 and Barbieri et al. (2016). The reason for using a different face perception task here was to equate the structure of the identity, object, and face perception tasks, to enable comparison across the tasks in the event of divergent stimulation effects, while also facilitating comparison across the two Experiments in this study. Moreover, in order to facilitate comparison across Experiment 1s and 2, we utilized the same stimulation parameters across the two Experiments where possible, this in turn resulted in further discrepancies with the methodology employed by Romanska et al. (2015). Notably, here we used 1.5 mA intensity (contrasting with 1 mA in Romanska et al.), and due to the capability of Neuroelectrics stimulator, the frequency range employed in the current study was 100–500 Hz. Romanska and colleagues administered up to 640 Hz (personal communication). Thus, it is possible that 1.5 mA intensity and/or the 100–500 Hz frequency range employed in the current study may account for the discrepant findings. Given these discrepancies, it will be important for future research to attempt a direct replication of the methodology employed by Romanska and colleagues to provide more conclusive evidence of the replicability of enhanced face perception following high-frequency tRNS.

One strength of the two experiments in the current study, compared to previous studies, was our more comprehensive investigation of potential demographic variables and baseline performance that could mask or inflate the effects of stimulation (Krause and Kadosh, 2014). Across both experiments, there was no evidence of group differences on a range of demographic variables (i.e., age, sex, IQ). There were also no significant group differences in baseline measures of face recognition ability and facial expression recognition across either study. While one obvious criticism may be that the baseline performance was assessed using tasks that were different from the tasks of interest in the study, this was considered important to avoid practice effects confounding performance on the tasks following stimulation. However, it remains possible that despite the absence of baseline differences on the CFMT and the Emotion Hexagon task, that there may have been meaningful differences between the groups on unmeasured variables that influenced performance on the primary tasks of interest, which may in turn have masked the effects of stimulation. Indeed, inter-individual variability is thought to not only influence the effects of transcranial electrical stimulation, but also interact with stimulation parameters, such as timing of stimulation (offline vs. online), electrode montage and current intensity (Fertonani and Miniussi, 2017).

The importance of such inter-individual variability in the current study is highlighted by a rather noteworthy (albeit non-significant) discrepancy in performance on the face perception task across the sham stimulation groups in Experiments 1 and 2. Theoretically, these two groups should be performing equivalently on the three tasks, however, if we consider performance on the face perception task alone, there is a 5% difference in performance accuracy (Experiment 1: *M* = 77; Experiment 2: *M* = 82), suggesting that inter-individual variability can account for considerable variability in performance. This is particularly meaningful when considering that the mean difference in performance of the active and sham stimulation groups on the face perception task in Barbieri et al. (2016) was only 7%.

One obvious alternative to address the limitation of inter-individual variability inherent in between-subjects designs is the use of within-subjects designs. While the nature of within-subjects designs means baseline individual differences are controlled for, there do remain other potential confounds that have the potential to mask effects of stimulation. Specifically, the influence of practice effects across sessions remains an issue, as the magnitude of practice effects invariably differ across individuals, providing the potential to mask or inflate stimulation effects, particularly where the practice related change in performance is greater than the magnitude of any change in performance resulting from stimulation.

In sum, the current results failed to replicate previous findings of enhanced face perception using anodal tDCS (Experiment 1) and high-frequency tRNS (Experiment 2) targeting the occipitotemporal cortex. These findings align with a growing body of studies that have failed to replicate previously reported enhancements or reported null results following administration of tDCS (e.g., Koenigs et al., 2009; de Hollander et al., 2016; Vannorsdall et al., 2016; Nilsson et al., 2017) and suggest different efficacy of, seemingly, related stimulation protocols. It will be important for future studies to explore the foundation of these divergent effects in greater detail.

## Data Availability Statement

All datasets analyzed for this study are included in Supplementary Files.

## Author Contributions

MW, RP, DR, and MN designed the study and wrote the manuscript. MW, DR, and AC, collected the data. MW, AC, and DR conducted the data analysis. All authors reviewed the manuscript.

## Conflict of Interest Statement

MN is member of the scientific advisory board of Neuroelectrics^©^. The remaining authors declare that the research was conducted in the absence of any commercial or financial relationships that could be construed as a potential conflict of interest.
